# Pseudotumoral form of soft tissue tuberculosis of the hand: six cases

**DOI:** 10.11604/pamj.2016.25.178.8918

**Published:** 2016-11-21

**Authors:** Mohamed Ali Sbai, Sofien Benzarti, Emna Chalbi, Hichem Msek, Adel Khorbi

**Affiliations:** 1 Orthopedic surgery and Trauma Department, Maamouri Hospital, Nabeul, Tunisia; 2 Anatomic pathology Department, Maamouri Hospital, Nabeul, Tunisia

**Keywords:** Tuberculosis, mycobacterium tuberculosis, soft tissue, pseudotumoral form, hand

## Abstract

Musculoskeletal involvement is not uncommon in extra-pulmonary tuberculosis, but the localization in the soft tissue of the hand is very rare. Diagnosis is much more difficult because of the atypical location and non-specific symptoms. We report 6 cases of pseudotumoral form of soft tissue tuberculosis of the hand treated in our department during the past 12 years. The mean age of the patients was 51 years with extremes of 44 and 63 years. A marked female predominance was observed (sex ratio = 0.2). All patients presented with swelling of the finger, two of which were painful swelling. All long fingers were involved; the thumb was involved in two cases. The histological study after excisional biopsy revealed caseating giant cell granulomas with epitheloid cells confirming the diagnosis. Antibacillary chemotherapy promoted healing and good outcome in our patients.

## Introduction

Tuberculous involvement is a rare entity in pathology of the hand; it could be expressed by osteitis, osteoarthritis or tenosynovitis [1, 2]. Involving the soft tissues in its pseudotumoral form is exceptional, often raises diagnostic issues and is usually incidentally discovered on histological findings [3]. In this study we present 6 cases of pseudotumoral form of soft tissue tuberculosis of the fingers, insisting on the rarity of this entity, the clinical presentations, the different therapeutic options and their outcome. A review of the literature was made.

## Methods

It is a retrospective study including 6 patients treated in the past 12 years in our department. All these patients presented with swelling of the finger and whose histological study after surgical excision revealed soft tissue tuberculosis. All these patients received both, surgical excision of the tumor and antituberculous chemotherapy. Our results were assessed according to the functional and aesthetic outcome.

**Patient consent and ethical approval:** Written informed consent was obtained from the patient for publication of this Case report and any accompanying images. A copy of the written consent is available for review if necessary. The study was approved by the institutional review board.

## Results

**Figure 1:**
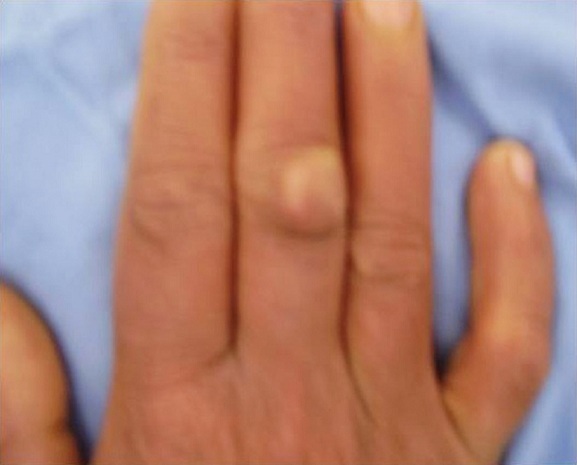
Soft tissue mass on the dorsal aspect of the middle finger of the right hand before excision

**Figure 2:**
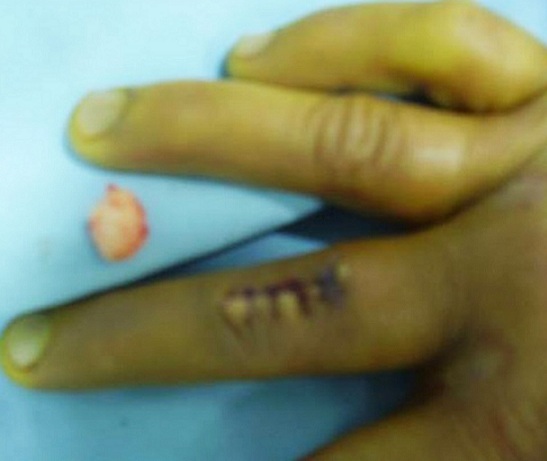
Soft tissue mass on the dorsal aspect of the middle finger of the right hand after excision

**Figure 3:**
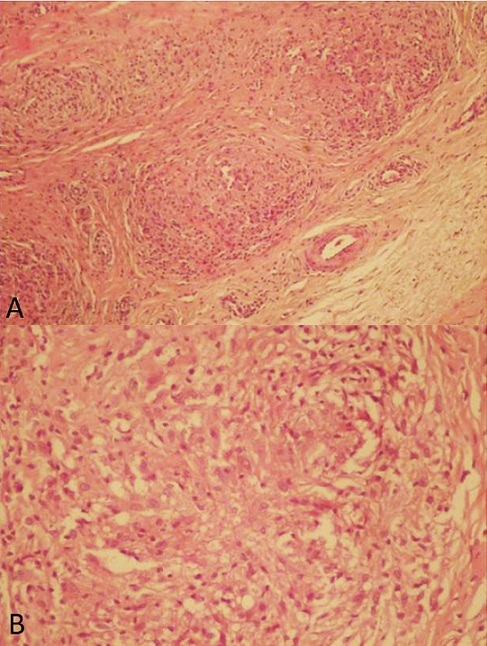
Histological study showing granuloma with caseous necrosis (hematoxylin and eosin stain, X10 (A), X40 (B))

**Figure 4:**
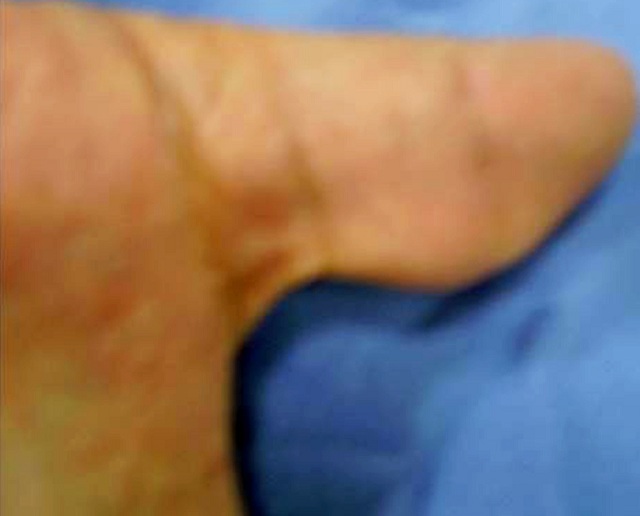
Pre-operative view of the soft tissue mass of the palmar aspect of the left thumb

**Figure 5:**
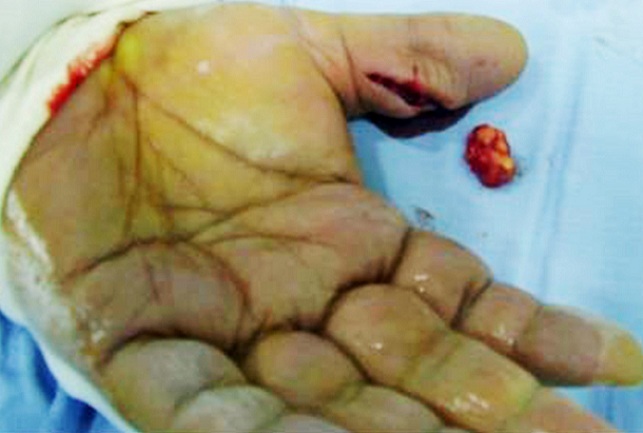
Intra-operative view of the soft tissue mass of the palmar aspect of the left thumb

**Figure 6:**
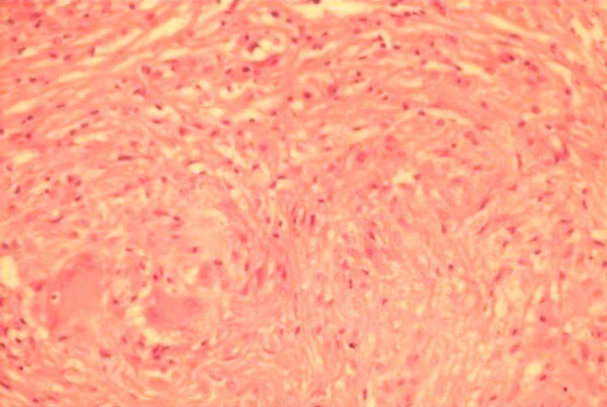
Histological study showing granuloma with caseous necrosis (Hematoxylin and eosin stain, X40)

**Figure 7:**
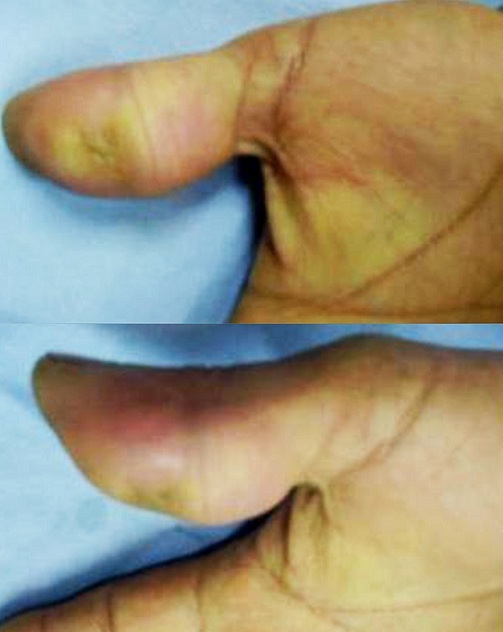
Pre-operative view of the soft tissue mass of the palmar aspect of the right thumb

Our series is made of six patients divided into five women and one man, the mean age is 51 years with extremes of 44 and 63 years, two patients are diabetic, one patient is hypertensive and only one patient had a prior history of pulmonary tuberculosis. Clinically, all the patients presented with a swelling of the soft tissues of the finger, it was painless, soft, relatively fixed to the deeper layers, of 1 cm diameter on average, evolving for 4 months on average without any inflammatory signs, evoking a benign tumor of the soft tissues of the hand (lipoma, sarcoidosis, sebaceous cyst). These tumors were located in the thumb in two cases and involving the long fingers in the other cases, the dominant hand was affected in 4 cases, the tumor was located on the palmar aspect of the finger in two cases and the dorsal aspect in the 4 other cases. In one case we recorded a double location in the same upper limb (thumb and forearm). Plain radiographs showed no bone injury, all patients underwent a chest radiograph to exclude healed or active pulmonary tuberculosis. Ultrasound performed in 3 patients showed very limited hyperechoic images. Laboratory tests noted an elevated erythrocyte sedimentation rate. An excisional biopsy was performed in all patients under regional anesthesia with axillary block. Direct examination showed acid fast bacilli on Ziehl-Neelsen stain in two cases. Culture on löwenstein-Jensen medium was positive in five cases. The histological study revealed a granulomatous lesion with caseating giant cells suggestive of tuberculosis in all cases. A general examination looking for other locations of tuberculosis was negative. All patients were treated with anti-tuberculous chemotherapy (ATC), using four drugs (rifampicin, isoniazid, pyrazinamide and ethambutol) for two months and two drugs (isoniazid and rifampicin) for 4 months associated to a rehabilitation of the hand for one month with good functional and aesthetic results (Table 1) (Figure 1, Figure 2, Figure 3, Figure 4, Figure 5, Figure 6, Figure 7).

**Table 1:** Epidemiological and clinical characteristics, management and results

Cases	age	sex	medical history	finger	side	seat	Other location	Bacteriology		Histology	treatment	result
								Direct exam	Culture			
Case 1	48	M	Pulmonary tuberculosis	middle	right	dorsal	-	Negative	positive	Caseous necrosis	6 months	good
Case 2	52	F	diabetic	thumb	left	palmar	forearm	Negative	positive	Caseous necrosis	6 months	good
Case 3	49	F	-	thumb	right	palmar	-	Positive	positive	Caseous necrosis	6 months	good
Case 4	63	F	hypertensive	ring	right	dorsal	-	Positive	-	Caseous necrosis	6 months	good
Case 5	52	F	diabetic	little	left	dorsal	-	Negative	positive	Caseous necrosis	6 months	good
Case 6	44	F	-	index	right	dorsal	-	Negative	positive	Caseous necrosis	6 months	good

## Discussion

The most common locations for extra-pulmonary tuberculous involvement are lymph nodes, intestine, genitourinary tract and musculoskeletal system [4]. Hand involvement is rare, Pseudotumoral form of soft tissue tuberculosis of the hand and wrist is exceptional. Only few cases were published; reviewing the large series of tuberculosis of hand and wrist, only Ben Kaddeche reported 3 cases of pseudotumoral form in the fingers (Table 2). To our knowledge, besides the localization in the fingers, no other localization in the hand was reported, but some cases of pseudotumoral form of soft tissue tuberculosis of the wrist were published [5–8]. Extrapulmonary tuberculosis (such as of the hand) usually result from reactivation of primary foci and secondary hematogenous spread. In the musculoskeletal system, the organisms are ingested by mononuclear cells that merge into epithelioid cells. A tubercle is formed when lymphocytes form a ring around a group of epithelioid cells. Caseation then occurs within the center of the tubercle [13]. Although in his study of 11 patients with tuberculosis of the wrist Benchakroun described active pulmonary tuberculosis in four cases [3], there was no case of active or treated pulmonary tuberculosis in our study. Histologically, active sites of tuberculous involvement are marked by a characteristic granulomatous inflammatory reaction that forms both caseating and non-caseating tubercles. Individual tubercles are microscopic; it is only when multiple granulomas unite that they become macroscopically visible. The granulomas are composed of central necrosis surrounded by epithelioid cells and lymphocytes, some of epithelioid cells fuse to form multinucleate giant cells [13]. They are usually manifested in a solitary lesion, multiple lesions are also possible (case3). Common symptoms of tuberculosis include low-grade fever, anorexia, weight loss, and night sweats. Specific symptoms and signs for the localization in the hand include pain, stiffness, swelling, joint effusion, digital enlargement, and carpal tunnel syndrome. There is usually a delay in the diagnosis of hand tuberculosis due to occult symptoms and slow progression, which frequently leads to more morbidity and a worse outcome [9]. There are no specific tests for preoperative diagnosis of the disease, which also leads to a delayed diagnosis. Laboratory findings are generally negative, except for the erythrocyte sedimentation rate (ESR), which is usually increased. Plain radiographs of tuberculosis of the short tubular bones of the hand show various findings such as cysts, lytic lesions, joint destruction, and periosteal reaction. Magnetic resonance imaging (MRI) is also a non-specific tool, but could better assess the extention of the lesion than plain radiography [9, 10]. MRI may show thickening of the synovial membrane with increased vascularization, fluid within the tendon sheath, reactive inflammation around the tendon, or swelling of the tendon. The thickened tenosynovium and synovium usually present as low signal intensity on T1 and T2-weighted images with enhancement after gadolinium administration that is suggestive of granuloma [11]. Confirmation is provided by the histological study and the research of Mycobacterium tuberculosis after culture on the Löwenstein-Jensen medium [12].

**Table 2:** Cases of pseudotumoral form of soft tissue tuberculosis of the hand published in the series of hand tuberculosis

Author	Year	Serie	Pseudotumoral form
Bush [4]	1984	11	0
Benkeddache [9]	1988	45	3
Bahri [1]	1998	23	0
Celester-Barreiro [10]	2005	11	0
Kotwal [11]	2009	32	0
Dlimi [12]	2011	30	0

## Conclusion

Pseudotumoral form of soft tissue tuberculosis of the hand is exceptional. Diagnosis is very difficult due to the atypical location and non-specific symptoms. It is usually incidentally discovery on histological findings. Surgical excision with anti-tuberculous chemotherapy provided good functional and aesthetic results.

### What is known about this topic

- In the absence of symptoms suggestive of pulmonary tuberculosis, the diagnosis of musculoskeletal tuberculosis is often delayed;
- Tuberculous involvement is a rare entity in pathology of the hand; it could be expressed by osteitis, osteoarthritis or tenosynovitis.

### What this study adds

- Tuberculosis involving the soft tissues of the hand in its pseudotumoral form is exceptional, but should be suspected especially in endemic areas;
- Diagnosis is very difficult due to the atypical location and non-specific symptoms. It is usually incidentally discovery on histological findings;
- Treatment consists in surgical excision with anti-tuberculous chemotherapy, usually providing good functional and aesthetic results.
